# Osteoarthritis is associated with symptoms of common mental disorders among former elite athletes

**DOI:** 10.1007/s00167-016-4255-2

**Published:** 2016-08-03

**Authors:** Nannet Schuring, Haruhito Aoki, Janine Gray, Gino M. M. J. Kerkhoffs, Mike Lambert, Vincent Gouttebarge

**Affiliations:** 1 Academic Center for Evidence-based Sports medicine (ACES), Academic Medical Center, Amsterdam, The Netherlands; 2 St. Marianna University School of Medicine, Kawasaki, Japan; 3 Division of Exercise Science and Sports Medicine, University of Cape Town, Cape Town, South Africa; 4 Department of Orthopaedic Surgery, Academic Medical Center, Amsterdam, The Netherlands; 5 Amsterdam Collaboration for Health & Safety in Sports (ACHSS), Academic Medical Center/VU Medical Center, Amsterdam, The Netherlands; 6 EMGO + Institute for Health and Care Research, VU University Medical Centre, Amsterdam, The Netherlands; 7 World Players’ Union (FIFPro), Scorpius 161, 2132 LR Hoofddorp, The Netherlands

**Keywords:** Osteoarthritis, Common mental disorders, Elite athletes, Quality of life, Mental health

## Abstract

**Purpose:**

The primary aim was to establish the association between osteoarthritis (OA) and the occurrence and comorbidity of symptoms of common mental disorders (CMD: distress, anxiety/depression, sleep disturbance, adverse alcohol use) in a group of former elite athletes (rugby, football, ice hockey, Gaelic sports and cricket). A secondary aim was to explore this association in the subgroups of sports.

**Methods:**

Cross-sectional analysis was performed on the baseline questionnaires from five prospective cohort studies conducted between April 2014 and January 2016 in former elite athletes of rugby, football, ice hockey, Gaelic sports and cricket. The presence of OA (diagnosed by a medical professional) was examined with a single question, and symptoms of CMD were evaluated through multiple validated questionnaires (4DSQ, GHQ-12, PROMIS, AUDIT-C).

**Results:**

There was a significant association between OA and symptoms of distress (OR 1.7, 95 % CI 1.2–2.6), sleep disturbance (OR 1.6, 95 % CI 1.1–2.3), adverse alcohol use (OR 1.8, 95 % CI 1.2–2.6) and a comorbidity of symptoms of CMD (OR 1.5, 95 % CI 1.0–2.1) in former elite athletes.

**Conclusion:**

OA might be a risk factor for developing symptoms of CMD in former elite athletes. The clinical relevance of this study is that an interdisciplinary approach to the clinical care and support of former elite athletes after their careers is advocated as the interaction between the physical and mental health issues occurring on the long term is complex. Monitoring OA among former elite athletes should be empowered while strategies to prevent symptoms worsening should be developed and implemented. The self-awareness, prevention and care of mental health problems that might occur after a professional sports career should also be addressed.

**Level of evidence:**

Level III.

## Introduction

Osteoarthritis (OA), the most common joint pathology worldwide, causes structural and functional failure of synovial joints with loss and erosion of the articular cartilage [3, 20]. The prevalence of OA (symptomatic and non-symptomatic) reaches up to 80 % of the Western European population over 75 years of age, and it is estimated that approximately 10 % of the world’s population of 60 years and older have physical and/or pain symptoms that can be attributed to OA [3, 29]. OA has major consequences for an individual’s functioning during daily activities: it is the tenth leading cause of disability in high-income countries, and it is expected to become the world’s fourth leading cause of disability by 2020 [10, 20, 25]. The socio-economic impact of OA is substantial with the total annual disease costs being estimated at $5700 per individual per year. OA is also responsible for losses in work, social activities and difficulties in performing self-care [4, 22, 26].

The impact of OA is not limited to physical symptoms only; several studies have shown that OA has a negative effect on a person’s mental well-being and their health-related quality of life [4, 13]. A recent US study measured the health-related quality of life of patients with OA using a generic quality of well-being scale. The quality of well-being scale score of these patients was 0.64, which was lower than that of the community-matched cohort (0.71) and similar to scores from patients with depression (0.64) or advanced cancer (0.63) [4, 17].

Two recent systematic reviews showed the prevalence of OA was high among former elite athletes. Among retired professional footballers, the prevalence of knee OA ranged between 40 and 80 %, and the prevalence of ankle OA between 12 and 17 % [24]. Among former elite athletes from various sport disciplines (ice hockey, basketball, handball, track and field athletes, power sport athletes, weight lifters, shooters and non-impact sport athletes), the prevalence of hip OA ranged from 2 to 60 %, and from 16 to 95 % for knee OA [12, 21]. OA in retired professional footballers causes pain, discomfort and functional limitations. It has been also suggested that OA could have an adverse impact on mental health: 37 % of the retired professional footballers suffering from OA reported moderate or severe problems related to anxiety/depression [11]. Recent studies have shown that symptoms of common mental disorders (CMD) such as distress, anxiety/depression, sleeping disturbance and adverse alcohol behaviour are largely reported after a career in elite sports. However, the extent of the association between OA and symptoms of CMD in former elite athletes has not been established yet [13, 15, 18].

Consequently, the primary aim of this study was to explore the association between OA and the occurrence and comorbidity of symptoms of CMD (distress, anxiety/depression, sleep disturbance, adverse alcohol use) among former elite athletes from rugby, football, ice hockey, cricket and Gaelic sports (football and hurling). The hypothesis was that former elite athletes suffering from OA were more likely to report symptoms of CMD than former elite athletes without OA. The secondary aim was to explore whether this association differed between the various subgroups of sports.

## Materials and method

The cross-sectional analyses were performed on the baseline questionnaires from five prospective cohort studies conducted among former elite athletes from the rugby, football, ice hockey, Gaelic sports (football and hurling) and cricket. The participants fulfilled the following inclusion criteria at recruitment: (I) age of 50 years or younger, (II) male, (III) able to read and comprehend texts fluently in either in English, French or Spanish, and (IV) a member of the national rugby union players’ associations, the national footballers’ unions, national ice hockey players’ associations, South African cricketers’ association or Gaelic players’ association from Finland, France, Ireland, Norway, South Africa, Spain, Sweden and/or Switzerland. In our study, the definition for a former elite athlete was that he had trained to improve performances, to have competed in the upper national division or league and to have had training and competition as major activity (way of living) or focus of personal interest, devoting several hours in all or most of the days for these activities, and exceeding the time allocated to other types of professional or leisure activities [2].

### Independent variable: osteoarthritis

The presence and location of OA diagnosed by a medical professional was examined through a single question (‘Have you been diagnosed with osteoarthritis by a medical professional?’). In the questionnaire, the following definition of OA was given to participants ‘Osteoarthritis is the damage of the joint’s cartilage that might lead to symptoms such as pain, stiffness or swelling in the given joint and that might impact functioning in either sport, work or daily life’.

### Dependent variables: symptoms of common mental disorders

#### Distress

Distress in the previous 4 weeks was measured using the distress screener (three items scored on a three-point scale), which is based on a four-dimensional symptom questionnaire (4DSQ) [14, 30]. This is a self-rating questionnaire and is used as a convenient tool to assess common psychosocial symptoms. An example of a question is ‘Have you recently felt tense?’. Possible answers are no (0 points), sometimes (1 point), regularly (2 points) or (very) often (2 points). The 4DSQ, has been validated in several populations and languages including English, French and Spanish (internal consistency: 0.6–0.7; test–retest coefficients: ≥0.9; criterion-related validity: area under ROC curve ≥0.8) [14, 30]. A total score ranging from 0 to 6 was obtained by summing up the answers on the three items, a score of 4 or more indicating the presence of distress [14, 30].

#### Anxiety/Depression

The 12-item General Health Questionnaire (GHQ-12) is a self-rating questionnaire designed for detecting individuals with a diagnosable psychiatric disorder related to anxiety/depression in the previous 4 weeks. The original version had 60 items, and these were reduced to 12 items [14]. An example of an asked question in this questionnaire is ‘Have you recently been able to enjoy your normal day-to-day activities?’ with the following possible answers: better as usual, same as usual, less than usual, much less as usual. This 12-items questionnaire is validated in several populations and languages including English, French and Spanish (internal consistency: 0.7–0.9; test–retest coefficients: 0.8; criterion-related validity: sensitivity ≥0.7, specificity >0.7, area under ROC curve ≥0.8) [14, 28]. Based on the traditional scoring system, a total score ranging from 0 to 12 was calculated by summing up the answers on the 12 items, with a score of 4 or more indicating the presence of anxiety/depression (0 for favourable answers, 1 for unfavourable answers) [14, 28].

#### Sleep disturbance

Based on the PROMIS (short form), sleep disturbance in the previous 4 weeks was assessed through four single questions scored on a four-point scale (0 for favourable answers, 1 for unfavourable answers) [14, 32]. An example of a given question ‘In the past 4 weeks, I had difficulty falling asleep’. Possible answers: not at all (0 points), a little bit (0 points), somewhat (0 points), quite a bit (1 point), very much (1 point). A total score ranging from 0 to 4 was obtained by summing up the answers to the four questions, a score of 2 or more indicating the presence of sleep disturbance. The PROMIS has been validated in several populations and languages including English, French and Spanish (internal consistency: >0.9; test–retest coefficients: 0.8; construct validity: product–moment correlations ≥0.9) (for detailed information, see www.nihpromis.org) [14, 32].

#### Adverse alcohol use

Level of alcohol consumption was assessed using the three-item AUDIT-C. The AUDIT-C test has been validated in several populations and languages including English, French and Spanish (test–retest coefficients: 0.6–0.9; criterion-related validity: area under ROC curve 0.70–<1.0) [8, 14]. An example of a question is ‘How often do you have a drink containing alcohol?’ with possible answers: never, once a month or less, 2–4 times a month, 2–3 times a week, 4 or more times a week [8, 14]. A total score ranging from 0 to 12 was obtained by summing up the answers on the three items, a score of 5 or more indicating the presence of adverse alcohol use [8, 14].

#### Comorbidity of symptoms

Comorbidity of symptoms of CMD was defined as having two or more simultaneous symptoms of CMD (distress, anxiety/depression, sleep disturbance and adverse alcohol use).

### Procedure

Based on the dependent and independent variables, an electronic anonymous questionnaire available in English, French and Spanish was compiled (FluidSurveys™). In addition, the following descriptive variables were retrieved: age, stature, body mass, duration of professional sports career, years since retirement, nature of retirement (voluntarily or not), current occupation and number of working hours per week. Information about the study was emailed to potential participants by their respective association involved in the study. The invitation procedures were blinded to the responsible researchers for reasons of privacy and confidentiality of the former elite athletes. The participants were asked to give their informed consent and to complete the online questionnaire within 2 weeks while reminders were sent after 2 and 4 weeks. The questionnaire took about 20 min to complete. Ethical approval of the study was provided by the board of St. Marianna University School of Medicine (2014/04/16; Kawasaki, Japan), the Faculty of Health Sciences Human Research Ethics Committee of the University of Cape Town (642/2014, 843/2014; Cape Town, South Africa) and the Medical Ethics Review Committee of the Academic Medical Center (W15_060#15.0072, W15_171#15.0207; Amsterdam, The Netherlands). The present study was conducted in accordance with the 2013 Declaration of Helsinki and its later amendments or comparable ethical standards.

### Statistical analysis

Data were analysed using the statistical software IBM SPSS Statistics 23.0 for OS X. Descriptive data analyses (mean, standard deviation, frequency, range) were calculated for all variables in this study, including the frequency of participants that reported OA and symptoms of CMD. Univariate logistic regression analyses (expressed as odds ratio OR and related 95 % CI) were used to explore whether former elite athletes who suffered from OA (dichotomous independent variable) were more likely to report (simultaneously) symptoms of CMD (dichotomous dependent variables) than former elite athletes who did not suffer from OA (non-causal association). Under the assumption of at least 58 participants within a particular sport, similar univariate logistic regression analyses were conducted within each subgroup for sports, which met this criterion [16]. In accordance with a sample size calculation for testing the relationship between independent and dependent variables (N > 50 + 8 *m* where *m* is the number of independent variables), sample size of at least 58 participants was needed [16]. With regard to the five subgroups, i.e. five sports involved in our study, we strived to recruit a total sample size of minimum 290 participants (minimum 58 per sport) [16].

## Results

### Participants

A total of 2218 former elite athletes were contacted to participate in this study (1230 in rugby, 307 in football, 420 in ice hockey, 180 in cricket, 81 in Gaelic sports), from which 624 gave their written informed consent and completed the online questionnaire: 295 former rugby players (47 %), 220 former football players (35 %), 61 former ice hockey players (10 %), 27 former cricket players (4 %) and 21 former Gaelic sport athletes (3 %). From those, 22 participants did not answer the question about OA, and their data were consequently discarded. The mean age of the respondents was 36.7 (SD 6.3) years. Participants had an elite sports career with a mean duration of 10 years (SD 5) and were retired for 6 (SD 5). Approximately 42 % did not end their careers as elite athletes voluntarily. About 60 % of the participants were working in a paid position for about 37 h a week. All characteristics of the participants are presented in Table 1.

**Table 1:** Characteristics of the participants

Variables	Overall	With OA (n = 200)	Without OA (n = 402)
Age (in years; mean ± SD)	37 ± 6	38 ± 5	36 ± 6
Height (in cm; mean ± SD)	183 ± 8	184 ± 7	183 ± 8
Weight (in kg; mean ± SD)	93 ± 17	94 ± 16	93 ± 17
Duration of sports career (in years; mean ± SD)	10 ± 5	11 ± 4	10 ± 5
Duration of retirement (in years; mean ± SD)	6 ± 5	7 ± 5	56 ± 5
Involuntary retired from sports (%)	42	44	42
Currently (self-) employed (%)	60	52	64
Working hours per week (mean ± SD)	37 ± 14	42 ± 12	42 ± 14
Prevalence of osteoarthritis (%)	33.2 %	–	–
*Prevalence of symptoms of CMD* (%)			
Distress	22.1	28.6	18.8
Anxiety/depression	27.1	30.8	25.3
Sleep disturbance	26.9	33.2	23.9
Adverse alcohol use	26.2	33.8	22.5
Comorbidity (≥2)	27.2	33.5	25.6

*SD* standard deviation, CMD symptoms, symptoms of common mental disorders, *OA* osteoarthritis; comorbidity, having two or more than two symptoms of common mental disorders

**Fig. 1:**
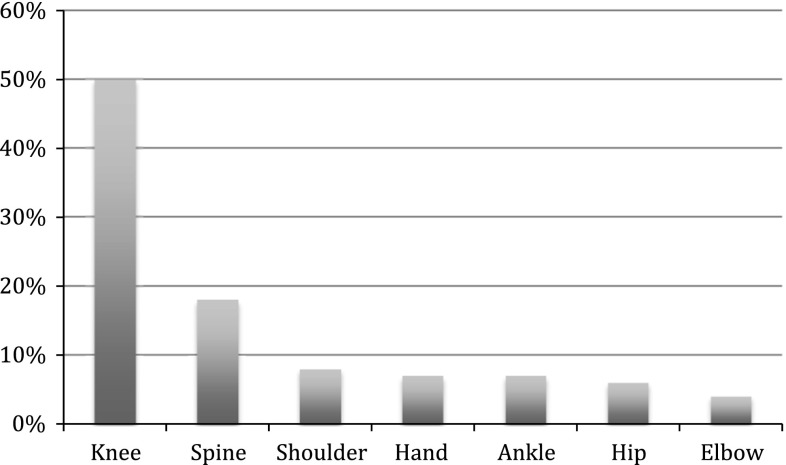
Distribution of osteoarthritis (OA) by anatomical site

**Fig. 2:**
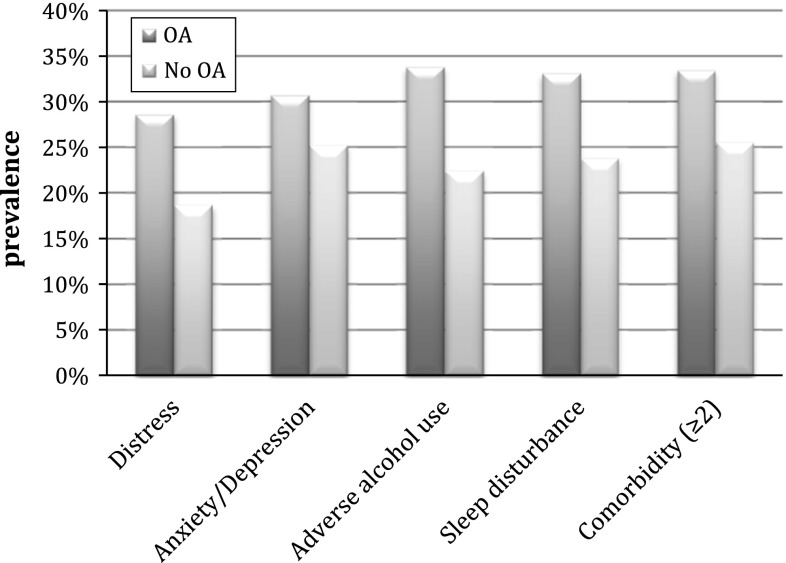
Prevalence of symptoms of common mental disorders among former elite athletes with and without OA

Around 33 % (N = 200) of the respondents reported that a medical professional diagnosed them with OA. The most reported anatomical site to have OA was the knee with 50 % of those diagnosed with OA reporting it in one or in both of their knee joints. The distribution of OA by anatomical site is presented in Fig. 1. The prevalence of symptoms of CMD ranged from 22 % for distress to 27 % for anxiety/depression. In the group of former elite athletes with OA, this range was from 29 to 34 % for distress and adverse alcohol use, respectively. In the group of former elite athletes without OA this ranged from 19 to 25 % for distress and anxiety/depression, respectively (Fig. 2).

### Association between osteoarthritis and symptoms of common mental disorders

OA was statistically (not causally) associated with distress (OR = 1.7, 95 % CI 1.2–2.6), sleep disturbance (OR = 1.6, 95 % CI 1.1–2.3), adverse alcohol use (OR = 1.8, 95 % CI 1.2–2.6), comorbidity ≥2 (OR = 1.5, 95 % CI 1.0–2.1). The relative strength of the relationship between OA and symptoms of CMD (including across sports) is presented in Table 2.

**Table 2:** Association (OR and 95 % CI) between osteoarthritis and symptoms of common mental disorders among former professional athletes and among former professional athletes from football, rugby and ice hockey

	Distress	Anxiety/Depression	Sleep disturbance	Adverse alcohol use	Comorbidity (2 ≥)
Total	**1.7 (1.2–2.6)**	1.3 (0.9**–**1.9)	**1.6 (1.1–2.3)**	**1.8 (1.2–2.6)**	**1.5 (1.0–2.1)**
Rugby	**2.7 (1.5–4.7)**	1.6 (0.9**–**2.7)	**2.4 (1.4–4.1)**	1.6 (0.9**–** 2.9)	**2.1 (1.2–3.5)**
Football	1.0 (0.5**–**2.1)	0.8 (0.4**–**1.5)	0.9 (0.4**–**1.6)	0.5 (0.3**–**1.1)	0.7 (0.3**–**1.4)
Ice hockey	**1.2 (1.1–1.4)**	**1.4 (1.2–1.8)**	**1.3 (1.1–1.6)**	**3.7 (2.2–6.9)**	**1.5 (1.2–2.0)**

Statistically significant values are given in bold

## Discussion

The most important finding of the present study is the significant association (non-causal) between OA and symptoms of distress, sleep disturbance, adverse alcohol use and comorbidity of symptoms of CMD among former elite athletes.

### Perspective of the findings

OA is a prevalent joint disease in the general population, ranging between 10 and 20 % in the male general population (from age 35 and above) in many countries [19, 23]. Among former elite athletes from various sport disciplines (ice hockey, basketball, handball, track and field athletes, power sport athletes, weight lifters, shooters and non-impact sport athletes), the prevalence of OA reaches up to 60 % for the hip and ranges from 16 to 95 % for the knee [12, 22, 23]. In our study, 33 % of the former elite athletes reported that a medical professional had diagnosed them with OA (mostly in the lower limbs), which is in accordance with the prevalence found in previous studies [9, 13, 24]. Also in our study, there was a significant association between OA and symptoms of CMD among the former elite athletes. Consequently, it seems necessary to promote the appropriate management of this joint disease in former sportsman, not only for its physical consequences such as pain, discomfort and functional impairments, but also for its consequences related to symptoms of CMD.

A previous cross-sectional study, investigated the prevalence of OA and anxiety and depression via questionnaires, which were sent to 515 former professional football players in the UK [31]. A total of 284 players participated by returning the questionnaire (response rate 55 %), from which 138 (49 %) reported a diagnosis with OA on at least one site [31]. Significantly more respondents with OA reported problems with anxiety/depression than those without OA: 37 % of the participants with OA reported problems on the questionnaire dimension anxiety/depression compared to 19 % of the participants of the group without OA (χ^2^ = 10.48, *df* = 1, *p* = 0.001) [31]. Similar results were found in our study; namely that 31 % of the former elite athletes with OA reported problems related to anxiety/depression compared to 25 % of the former elite athletes without OA. As far as we know, this cross-sectional study is the first international study to explore the association of OA and symptoms of CMD among a large group of former elite athletes from different sports.

### Implications of the study

Our study confirms that both OA and symptoms of CMD are two major health problems that are prevalent among former athletes. This impacts on the long-term health consequences of a career in the elite sport. Our study also shows that OA and its related physical consequences (pain, discomfort, impairments) may be seen as a risk factor for the occurrence of symptoms of CMD. In a previous study, we advocated for an interdisciplinary approach to the clinical care and support of current professional athletes during their career [14]. The present study justifies such an interdisciplinary approach among former elite athletes after their careers, because the interaction between the physical and mental health issues occurring on the long term is complex.

The preventive and supportive measures for reducing OA or symptoms of CMD for former elite athletes are lacking. As previously advocated by active and former professional athletes as well as by sports physicians, the development of self-management interventions directed towards OA and symptoms of CMD should be explored [1]. Self-management strategies for chronic physical and mental health conditions, such as rheumatic diseases, diabetes and depression, are effective. These self-management strategies aim at engaging and promoting a healthy and active behaviour of patients by covering aspects such as self-awareness (information provision and patient education) and cognitive and behavioural therapy [5, 6, 27]. Another potential step forward in the medical care and support of former elite athletes is the development and implementation of an exit-career medical assessment (ExCMA). Such an ExCMA aims to raise self-awareness (information provision) about the long-term consequences of an elite sport career and counselling especially about OA’s remission, coping skills related to symptoms of CMD and promotion of healthy life style would be beneficial. Such an ExCMA is intended to be tested in professional football by the World Players’ Union (FIFPro) before a larger implementation across sports.

Our study has a number of limitations. Firstly, a cross-sectional design was used. Such a design allowed us to explore the association between OA and symptoms of CMD, but it did not allow any causal relationships to be established. It remains unknown when the former elite athletes were diagnosed with OA and if they have coped with having symptoms of CMD on previously in their life. A longitudinal study design following a group of elite athletes through their careers until several years after retirement is needed to confirm this observation. Secondly, the recruitment procedures were blinded for the researcher for privacy and confidential reasons. Consequently, it was not possible to conduct a non-response analysis. Lastly, an additional aspect worth discussing is that the participants were from different countries and cultures. It would be possible that there is a difference in the frankness about answering questions about symptoms of CMD based on the social desirability. This heterogeneous sample might have implications for determining the true prevalence symptoms of CMD in former elite athletes.

Several main strengths of our study can be acknowledged. First, the topic being studied, namely the long-term consequences of an elite sports career, is exponentially under scrutiny in medical sciences. Second, a large group of former elite athletes retired from rugby, football, ice hockey, Gaelic sports and cricket was secured, which provided a unique and innovative insight in this special population. Third, the assessment of patient-reported outcomes measures (PROMs) by validated questionnaires gives us real-time information about each participant having symptoms of CMD. If PROMS would be systematically used during and after a career in elite sports, it could be of great additional help for clinicians to easily identify relevant information, about the condition of the mental health of the elite athlete and for elite athletes themselves this could empower the clinicians in their clinical decisions and the athlete in seeking help [7].

The clinical relevance of this study is that an interdisciplinary approach to the clinical care and support of former elite athletes after their careers is advocated as the interaction between the physical and mental health issues occurring on the long term is complex. Our findings contribute to advancing knowledge about the long-term consequences of a career in elite sports. With more knowledge about the medical issues that occur after a career in sport, specific preventive and supportive measures can be developed and implemented to empower the durable health of former elite athletes.

## Conclusion

OA might be a risk factor for developing symptoms of CMD in former elite athletes. Therefore, monitoring OA among former elite athletes should be empowered while strategies to prevent symptoms worsening should be developed and implemented. The self-awareness, prevention and care of mental health problems that might occur after a professional sports career should also be addressed.
